# *Helicobacter pylori* Infection in Patients Undergoing Upper Endoscopy, Republic of Georgia

**DOI:** 10.3201/eid1503.080850

**Published:** 2009-03

**Authors:** Nato Tarkhashvili, Rusudan Beriashvili, Neli Chakvetadze, Maia Moistsrapishvili, Maia Chokheli, Merab Sikharulidze, Lile Malania, Nato Abazashvili, Ekaterine Jhorjholiani, Marina Chubinidze, Nanuli Ninashvili, Tamar Zardiashvili, Ucha Gabunia, Dimitri Kordzaia, Paata Imnadze, Jeremy Sobel, Jeannette Guarner

**Affiliations:** Centers for Disease Control and Prevention, Atlanta, Georgia, USA (N. Tarkhashvili, J. Sobel); National Center for Disease Control and Medical Statistics, Tbilisi, Republic of Georgia (R. Beriashvili, N. Chakvetadze, M. Moistsrapishvili, M. Chokheli, M. Sikharulidze, L. Malania, N. Abazashvili, E. Jhorjholiani, M. Chubinidze, N. Ninashvili, T. Zardiashvili, U. Gabunia, D. Kordzaia, P. Imnadze); Emory University School of Medicine, Atlanta (J. Guarner); Children's Healthcare of Atlanta, Atlanta (J. Guarner)

**Keywords:** Heliobacter pylori, ulcers, endoscopy, gastric cancer, gastritis, peptic ulcer disease, Republic of Georgia, letter

**To the Editor:** *Helicobacter pylori* infection is the principal cause of chronic active gastritis and peptic ulcer disease and a major contributor for gastric adenocarcinoma and mucosa-associated lymphoid tissue lymphoma (*1*). Approximately 50% of the world’s population is infected (*2*), but only 10%–20% of infected persons become symptomatic (*3*). The annual incidence rate of *H. pylori* infection is ≈4%–15% in developing countries, compared with ≈0.5% in industrialized countries (*4*). Studies in the Republic of Georgia (ROG), a developing country with an economy in transition, suggested that >70% of adults are infected with *H. pylori* (*5*,*6*) and that the prevalence rate of gastric cancer is 18 cases per 100,000 population, ≈6- to 9-fold higher than in the United States (National Center for Disease Control, Tbilisi, ROG, unpub. data, 2003). We investigated the prevalence of infection in patients in ROG in whom gastritis, peptic ulcer, and gastric cancer had been diagnosed.

We performed a cross-sectional study of patients referred for upper endoscopy from all regions of ROG to 23 tertiary-care medical centers in the capital, Tbilisi, during 2003–2005. Patients whose medical records and gastric biopsy slides were available were eligible for inclusion. Two pathologists reviewed hematoxylin and eosin–stained slides prepared from formalin-fixed, paraffin-embedded gastric biopsy specimens. Pathologists graded the amounts of *H. pylori*, acute and chronic inflammation, intestinal metaplasia, and atrophy according to the visual analogue scale of the Updated Sydney Classification System for Gastritis (*7*). Histologic characteristics were dichotomized as presence (grades >1) or absence (grade = 0) of a feature.

We conducted statistical analyses in SAS version 9.0 (SAS Institute, Inc., Cary, NC, USA). The human subjects committees at the National Center for Disease Control and Medical Statistics of ROG and the Centers for Disease Control and Prevention (Atlanta, GA, USA) approved the study.

We identified 90 eligible persons. Their median age was 62 years (range 6–81 years); 48 (54%) were male. Biopsy specimens were taken from the antrum in 89 (99%) persons and from the corpus in 1 person. *H. pylori* infection was diagnosed in 59 (72%) persons, acute inflammation in 81 (90%), chronic inflammation in 77 (87%), metaplasia in 29 (35%), and atrophy in 11 (16%). *H. pylori* was detected in 78% of patients who had gastritis, in 58% of patients who had peptic ulcer, and in 58% of patients who had dysplasia or gastric cancer (Table).

**Table:** Histopathologic characteristics assessed in the biopsy specimens of study participants, by final pathologic diagnosis, Republic of Georgia, 2003–2005.

Final pathologic diagnosis	No. *Heliobacter pylori* positive/no. tested (%), n = 82	No. with acute inflammation/no. tested (%), n = 90	No. with chronic inflammation/no. tested (%), n = 89	No. with intestinal metaplasia/no. tested (%), n = 84	No. with glandular atrophy/no. tested (%), n = 68
Gastritis* (n = 62)	45/58 (78)	55/62 (89)	55/62 (89)	19/60 (32)	10/50 (20)
Peptic ulcer (n = 14)	7/12 (58)	14/14 (100)	12/13 (92)	5/13 (38)	1/10 (10)
Dysplasia or cancer (n = 14)	7/12 (58)	12/14 (86)	10/14 (71)	5/11 (45)	0/8 (0)
Total (n = 90)	59/82 (72)	81/90 (90)	77/89 (87)	29/84 (35)	11/68 (16)

*According to the Updated Sydney Classification System for Gastritis (*7*).

In a multivariable Poisson regression model, *H. pylori* positivity was strongly associated with acute inflammation (adjusted prevalence ratio [aPR] 1.4, 95% confidence interval [CI] 1.2–1.8) and chronic inflammation (aPR 1.5, 95% CI 1.2–1.9). Age >50 years (aPR 0.9, 95% CI 0.8–1.2) and male sex (aPR 1.0, 95% CI 0.9–1.2) did not confer increased risk for *H. pylori* infection.

*H. pylori* requires gastric mucus for growth, and mucus produced by the metaplastic and neoplastic cells is postulated to lack characteristics that sustain growth of *H. pylori*. When *H. pylori* has been observed in patients with ulcers, intestinal metaplasia, and adenocarcinoma, the bacteria usually are present in areas of the stomach that do not have these lesions.

In this cohort of patients, 14 (16%) had dysplasia or adenocarcinoma. Dysplasia and eventually cancer occur in a small group of susceptible persons with atrophy and intestinal metaplasia (*8*,*9*). Thus, *H. pylori* is now considered a type-1 carcinogen, and the absence of infection is predicted to result in at least 60% fewer cases of gastric cancer worldwide (*10*).

Our study has several limitations. First, because of the study’s retrospective nature, pathologists reviewed archived hematoxylin and eosin–stained sections of biopsy specimens. The inability to use additional techniques (e.g., special staining, immunohistochemistry) commonly used to visualize *H. pylori* might have resulted in underdetection of the true rate of infection in the study population. Second, in 99% of cases, only 1 biopsy specimen was obtained from the antrum instead of the 5 antrum and corpus biopsy specimens recommended for appropriate diagnosis by the Updated Sydney Classification System (*7*). Third, the relatively small sample size precluded exploration of additional relationships between demographic characteristics and histologic grades.

Our results indicate a high prevalence of *H. pylori* in patients with gastritis, peptic ulcer, and gastric cancer and suggest that *H. pylori* infection represents a serious public health problem in ROG. Studies are needed to explore demographic, socioeconomic, and behavioral risk factors that contribute to the high prevalence of *H. pylori* infection in symptomatic and asymptomatic persons living in ROG so that preventive measures can be identified.
